# Ascending Aortic Wall Cohesion: Comparison of Bicuspid and Tricuspid Valves

**DOI:** 10.1155/2012/180238

**Published:** 2012-09-05

**Authors:** Jaroslav Benedik, Kevin Pilarczyk, Daniel Wendt, Jiri Indruch, Radek Flek, Konstantinos Tsagakis, Savvas Alaeddine, Heinz Jakob

**Affiliations:** ^1^Department of Thoracic and Cardiovascular Surgery, West-German Heart Center, University Hospital Essen, University of Duisburg-Essen, Hufelandstraße 55, 45147 Essen, Germany; ^2^Oprox a.s. Renneska trida 413/35, 63900 Brno, Czech Republic; ^3^Aortix a.s. Jugoslavska 144, 61300 Brno, Czech Republic; ^4^Department of Anesthesiology and Intensive Care Medicine, University Hospital Essen, University of Duisburg-Essen, Germany

## Abstract

*Objectives*. Bicuspid aortic valve (AV) represents the most common form of congenital AV malformation, which is frequently associated with pathologies of the ascending aorta. We compared the mechanical properties of the aortic wall between patients with bicuspid and tricuspid AV using a new custom-made device mimicking transversal aortic wall shear stress. *Methods*. Between 03/2010 and 07/2011, 190 consecutive patients undergoing open aortic valve replacement at our institution were prospectively enrolled, presenting either with a bicuspid (group 1, *n* = 44) or a tricuspid (group 2, *n* = 146) AV. Aortic wall specimen were examined with the “dissectometer” resulting in nine specific aortic-wall parameters derived from tensile strength curves (TSC). *Results*. Patients with a bicuspid AV showed significantly more calcified valves (43.2% versus 15.8%, *P* < 0.001), and a significantly thinner aortic wall (2.04 ± 0.42 mm versus 2.24 ± 0.41 mm, *P* = 0.008). Transesophageal echocardiography diameters (annulus, aortic sinuses, and sinotubular junction) were significantly larger in the bicuspid group (*P* = 0.003, *P* = 0.02, *P* = 0.01). We found no difference in the aortic wall cohesion between both groups as revealed by shear stress testing (*P* = 0.72, *P* = 0.40, *P* = 0.41). *Conclusion*. We observed no differences of TSC in patients presenting with tricuspid or bicuspid AVs. These results may allow us to assume that the morphology of the AV and the pathology of the ascending aorta are independent.

## 1. Introduction

Bicuspid aortic valve (AV) is the most common congenital aortic valve malformation, with a prevalence of 0.5–2% in the general population [1–3]. In case of AV stenosis or regurgitation, patients presenting with bicuspid AV may become symptomatic at a younger age, as compared to patients with a tricuspid AV [4]. In addition, bicuspid AVs are frequently associated with different aortic wall disorders, which may lead to aortic aneurysms or in the worst case to dissection or rupture. The dilatation of the aorta is often accompanied with abnormal histological findings in the aortic media, such as increased elastin fragmentation, decreased thickness, and increased distance of the elastic lamellae [5]. Another factor, which may influence aortic wall stability and the de novo aneurysm creation in patients presenting with a bicuspid AV, is the higher activity of the matrix metalloproteinases (MMP) 2 and 9 [6]. However, the relationship between bicuspid AVs and aortic wall properties—especially in case of normal aortic wall dimensions—has not been investigated in detail so far.

We have recently introduced a new-patented “Dissectometer” device, which is used to test the cohesion of the aortic wall [7]. The aim of the present study was therefore to compare the cohesion of the native aortic wall between patients undergoing aortic valve replacement with anatomically bicuspid and tricuspid AVs.

## 2. Material and Methods 

### 2.1. Study Design

The present study was approved by the Institutional Review Board and patients gave written informed consent. The study was a single-center, nonrandomized, including 190 consecutive patients who underwent aortic valve replacement at the West-German Heart Center Essen between March 2010 and July 2011. A total of 44 patients (group 1) showed a bicuspid AV and 146 patients a tricuspid AV (group 2). Aortic and bulbus dimensions were assessed by intraoperative transoesophageal echocardiography (TOE). Patients requiring additional procedures like concomitant valve or CABG surgery were included into the present evaluation. 

### 2.2. Operative Technique

All operations were carried out through a standard median sternotomy or partial upper sternotomy with cardiopulmonary bypass (CPB) with ascending aorta cannulation. TOE was performed prior to CPB. A transverse aortotomy was performed and a sample of the aortic wall was harvested from the edge of the aortic incision site and was immediately placed in cold saline until the cohesion test was performed. The pathology of the AV was classified (bicuspid or tricuspid and the severity of calcification was graded: grade 0—no, grade 1—mild, grade 2—moderate and grade 3—severe calcification. 

### 2.3. Intraoperative Echocardiography

TOE was performed prior to CPB in all patients. TOE was performed with a multiplane 2.9–6.7 MHz (6T-RS) phased-array-probe (Vivid i, GE Healthcare, Milwaukee, WI, USA). All aortic dimensions (diameter of the aortic annulus, aortic sinuses, sinotubular junction, and ascending aorta) were measured. 

### 2.4. Aortic Wall Cohesion Testing

**Figure 1:**
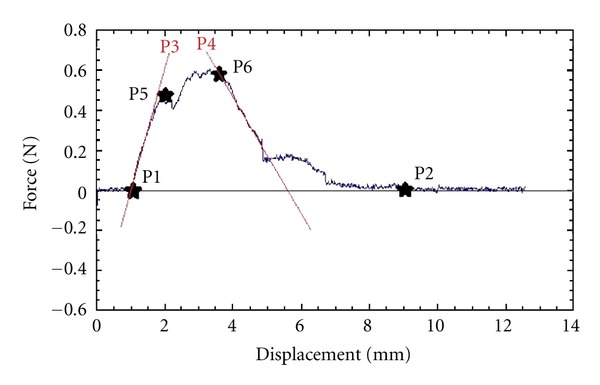
Tensile strain curve: localization of the points “asterisk” P1, P2, P5, and P6.

The time interval between harvesting off the sample and the final test did not exceed 2 hours (the majority of the tests were performed during the operation and the results could be obtained within 10 minutes from harvesting). Aortic wall cohesion testing was performed using the “Dissectometer” as previously described [7]. The results of the dissecting process were visualized as tensile strain curves (TSC), which were subsequently converted to numerical parameters as exemplified in Figure 1. P1 is the beginning of the positive deviation—the point when the dissectometer registers the tension in the sample. This point corresponds to the thickness of the sample. P2 is the point of the dissection and the power has a zero value. P5 is the first power maximum (in this point the power decreases temporarily). After this point the aortic wall sample is damaged irreversibly. P6 represents the “dissection limit” after which the power necessary to disrupt the aorta decreases. Usually P6 is higher than P5. P3 is the angle of the line between P1 and P5. This characteristic describes the elasticity of the aortic wall—the sharper the angle, the higher is the elasticity of the aorta. P4 is the angle of power decrease, which characterizes the cohesion of the aortic wall. P7 represents the area under the TSC which describes the total coherence of the aorta. These seven parameters were used to mathematically derive the next two parameters, P8 and P9. P8 is described as the “dissection tendency” (calculated as the maximal force divided by the downward angle) and P9 as the “dissection potential” (calculated as the sum of P8 and the square root of P7 divided by ten). Earlier studies revealed that the parameters P1–P6 are mostly descriptive parameters and P7–P9 are parameters with the capability to predict an unstable aortic wall as they show a highly significant correlation to histological signs of aortic wall instability with specificity and sensibility to predict aortic wall instability [7]. All cohesion tests were performed and analyzed by one observer blinded to all patients' data including aortic valve pathology.

### 2.5. Statistics

Descriptive statistics are summarized for categorical variables as frequencies (%). Continuous variables were reported as mean ± standard deviation. Groups were compared using Pearson's Chi-square or Fisher exact tests or Students' *t*-test as appropriate. A *P* value less than 0.05 was considered to indicate statistical significance. All statistical analyses were performed using the SPSS System, version 19.0 (IBM Corp., Armonk, NY, USA).

## 3. Results

Demographics and preoperative characteristics of both groups are listed in Table 1. Out of 190 patients, 44 patients presented with a bicuspid AV whereas 146 patients had a tricuspid AV. Patients with bicuspid AVs were significantly younger than patients with tricuspid AVs; male gender was predominated in both groups. Arterial hypertension was more frequently observed in the group of tricuspid AV whereas the preoperative distribution of other cardiovascular risk factors did not differ between both groups. The incidence of other comorbidities such as COPD or CAD was comparable between two groups without statistical significant difference. 

The echocardiographic and the dissectometer-derived results are summarized in Tables 2 and 3. In both groups the predominant underlying pathology of the AV was calcified stenosis. The degree of calcification was significantly higher in group 1 (19 patients 43.2%; 23 patients 15.8%  *P* < 0.001). Congruently, noncalcified valves were more frequent in group 2 (63 patients 43.2%; 10 patients 22.7%  *P* = 0.015). Acute aortic dissection was more common in the tricuspid AV group but the difference was too small to be considered of statistical significance. Patients of group 1 showed a significantly thinner aortic wall (2.04 ± 0.42 mm versus 2.24 ± 0.41 mm, *P* = 0.008). All diameters describing the dimension of the aorta in TOE (i.e., the annulus, aortic sinuses, sinotubular junction, and ascending aorta) were larger in group 1 (the difference in the diameter of the ascending aorta was too small to be considered to be statistical significant) Figure 1. Moreover, we did not observe any difference in the aortic wall cohesion between both groups as shown by the dissectometer parameters P7 (168.0 ± 85.6 versus 162.5 ± 90.6, *P* = 0.72), P8 (3.59 ± 2.02 versus 3.29 ± 2.12,  *P* = 0.40), and P9 (4.84 ± 2.28 versus 4.51 ± 2.33,  *P* = 0.41).

## 4. Discussion

A bicuspid AV is the most common congenital form of AV malformation. It is well established that a bicuspid valve is predisposing to result in aortic valve dysfunction such as stenosis or regurgitation. In addition, the body of evidence is growing that bicuspid AVs are accompanied with an increased risk of aortic wall pathology such as an aortic aneurysm or dissection. Currently, there are several possible explanations for this phenomenon. A bicuspid AV is often combined with connective tissue disorders and a higher fibrillin degradation which is reflected by an increased-MMP activity [6]. Moreover, each bicuspid AV morphologic group—left-noncoronary (L-N), right-left (R-L), and right-non-coronary (R-N)—possess unique signatures of matrix metalloproteinases (MMPs) and endogenous tissue inhibitors of metalloproteinases (TIMPs) [8]. 

A recently published study showed that in patients presenting with bicuspid AV, plasma concentrations of *α*1AT were higher in those patients with ascending aortic dilatation compared to the nondilated group [9]. Patients with bicuspid AV and a rapid progression of aortic dilatation have a more extensive cardiovascular risk profile including higher blood glucose levels, a higher incidence of coronary artery disease, more tobacco use, and a higher National Heart, Lung and Blood Institute 10-year risk of developing coronary heart disease. These observations may lead to the assumption that atherosclerosis plays an important role in the development of aortic dilatation in patients with bicuspid AV [10]. Accordingly, patients with stenotic bicuspid AVs are characterized by an increased macrophage infiltration and a higher degree of neovascularization when compared with patients presenting tricuspid AVs. However, it is unclear if these mechanisms also contribute to the increased risk of aortic dilatation [11]. As shown in a study by Yasuda et al., early elective aortic valve replacement in patients with bicuspid AV, with the aorta left untouched will not prevent future aortic dilatation [12]. This result supports the hypothesis of a large independence of aortic valve and ascending aortic pathology confirmed by the observation that patients with bicuspid AV disease can develop ascending aortic aneurysms without AV dysfunction. In addition, the growth rate of aortic aneurysms was significantly higher in patients with unreplaced bicuspid AVs compared to patients with tricuspid AVs [13]. However, this has been shown to be not associated with an increased number of aortic events [14]. In the present study, the incidence of aortic pathologies including acute aortic dissection or aortic aneurysm formation was comparable between the study groups although aneurysms were observed slightly more frequently in the tricuspid AV patients.

Furthermore, we observed that the cohesion of the aortic wall tested with the dissectometer was not dependent on the morphology of the AV. All three parameters that proved to have a good predictive power to detect aortic wall stability were comparable between the two study groups. Consistently with the literature, the bicuspid AV patients have developed aortic valve disorders at a younger age than the tricuspid AV patients and were predominantly males in our study.

It is unclear whether the type of fusion (real anatomy of the aortic valve) could influence aneurysm formation. Russo et al. combined type A fusion (right with left leaflet) with more severe aortic degeneration, however a some degree of degeneration was found in all patients [4]. Schaefer et al. described a larger diameter and a higher stiffness index in patients with type A fusion, which further supports these findings [15].

Based on morphometric analysis, patients with bicuspid AVs have a thinner media with a greater distance between the elastic lamellae [5]. This statement corresponds to our findings. Compared to patients with morphological tricuspid AVs, patients with bicuspid AVs had significantly thinner aortic walls. Our study showed that the thickness of the aortic wall is inversely proportional to its strength; however this difference was not of statistical significance. 

Biner et al. demonstrated that individuals with bicuspid AVs present significantly larger aortic valve annulus, bulbus, and diameters of the ascending aorta compared to individuals with tricuspid AVs [16]. These findings could be confirmed in our study. Ikonomidis et al. proposed MMPs and its inhibitors as prognostic markers in patients with aneurysm formation [17]. It is however unknown whether the congenital malformation of the AV leads to the development of an aortic aneurysm. La Canna et al. showed that pathology of the AV and aorta were independent [18]. This was supported with an equal rate of aneurysm growth and a low occurrence of the aortic events in patients with bicuspid AVs as compared to patients with tricuspid AVs. 

Although the incidence of ascending aneurysm was comparable in both groups, aortic dilation was observed more frequently in the aortic root, sinotubular junction, and ascending aortic segments in group 1, which is in accordance to the current literature [19]. 

## 5. Conclusion

Analysis of TSC curves of patients with tricuspid and bicuspid AVs in our study did not prove statistically significant differences between both groups. These results may allow us to assume that the morphology of the AV and the pathology of the ascending aorta are independent. However, this has to be proved in a larger amount of patients with longer follow-up. The comparison of the TSCs with histological examination and its correlation with MMP levels is part of an oncoming study.

**Limitation**

The present study was performed at a single tertiary care medical center with a relatively small sample size. Furthermore, continued long-term follow-up in patients with bicuspid AV who received surgical aortic valve replacement without replacement of the ascending aorta has to be done and will determine if the entity of a preoperative bicuspidalization of the AV will lead to future pathologies of the ascending aorta.

## Figures and Tables

**Table 1:** Demographics.

*n* = 190	Group1 (*n* = 44)	Group 2 (*n* = 146)	*P* value*
Age (years)	62.2 ± 12.1	69.0 ± 9.9	0.001
Female	10 (22.7%)	54 (37.0%)	0.08
BMI (kg/m^2^)	27.0 ± 3.6	27.7 ± 4.6	0.40
Height (cm)	172.7 ± 7.9	170.6 ± 9.5	0.18
Hypertension	30 (68.2%)	129 (88.4%)	0.003
DM	2 (4.5%)	23 (15.8%)	0.05
Renal insufficiency	6 (13.6%)	19 (13.0%)	0.92
Hypercholesterolemia	18 (40.9%)	66 (45.2%)	0.62
COPD	7 (15.9%)	18 (12.3%)	0.54
CAD	17 (38.6%)	64 (43.8%)	0.54

Data are presented as mean ± SD or number (%); BMI: body mass index; DM: diabetes mellitus; COPD: chronic obstructive pulmonary disease; CAD: coronary artery disease; *group 1 versus group 2.

**Table 2:** Underlying pathology.

*n* = 190	Group 1 (*n* = 44)	Group 2 (*n* = 146)	*P* value*
Aortic stenosis	23 (52.3)	61 (41.8)	0.22
Aortic insufficiency	9 (20.5)	51 (34.9)	0.07
Combination AS + AI	11 (25.0)	19 (13.0)	0.06
Ascending aneurysm	18 (40.9)	48 (32.9)	0.33
Aortic root dilatation	2 (4.5)	3 (2.1)	0.37
Marfan syndrome	1 (2.3)	0	0.07
Dissection	1 (2.3)	9 (6.2)	0.31
Calcification 0	10 (22.7)	63 (43.2)	0.02
Calcification 1	4 (9.1)	17 (11.6)	0.64
Calcification 2	11 (25.0)	43 (29.5)	0.57
Calcification 3	19 (43.2)	23 (15.8)	0.001

Data are presented as number (%); AS: aortic stenosis; AI: aortic insufficiency; *group 1 versus group 2.

**Table 3:** Transesophageal dimensions and TSC results.

*n* = 190	Group 1 (*n* = 44)	Group 2 (*n* = 146)	*P* value*
Aortic wall thickness (mm)	2.04 ± 0.42	2.24 ± 0.41	0.008
Aortic annulus (mm)	25.8 ± 3.3	24.2 ± 2.2	0.003
Aortic sinuses (mm)	38.1 ± 8.8	34.5 ± 7.9	0.02
Sinotubular junction (mm)	36.2 ± 10.4	31.8 ± 8.8	0.01
Ascending aorta (mm)	41.5 ± 12.0	37.9 ± 11.3	0.09
P7	168.0 ± 85.6	162.5 ± 90.6	0.72
P8	3.59 ± 2.02	3.29 ± 2.12	0.40
P9	4.84 ± 2.28	4.51 ± 2.33	0.41

Data are presented as mean ± SD; TSC: tensile strain curves; *group 1 versus group 2.
